# Prevalence and clinical correlates of psychotic symptoms in first-episode untreated female chinese patients with major depressive disorder

**DOI:** 10.1186/s12888-023-05011-4

**Published:** 2023-07-28

**Authors:** Ruijie Peng, Xiaobin Zhang, Ronghua Li, Guangya Zhang, Yan Yue, Siqi Wu, Yuxuan Wu, Ruchang Yang, Yue Zhou, Xiangdong Du, Xiangyang Zhang

**Affiliations:** 1 Medical College of Soochow University, Suzhou, China; 2 Institution of Suzhou Psychiatric Hospital, The Affiliated Guangji Hospital of Soochow University, Suzhou, 215131 China; 3 School Psychology and Mental Health, North China University of Science and Technology, Qinhuangdao, China; 4 Xuzhou Medical University, Xuzhou, China; 5 CAS Key Laboratory of Mental Health, Institute of Psychology, Chinese Academy of Sciences, 16 Lincui Road, Chaoying District, Beijing, 100101 China

**Keywords:** Depression, Females, First episode, Psychotic depression, Suicide attempt, Thyroid dysfunction

## Abstract

**Background:**

Recent studies have reported that psychotic symptoms are common in patients with major depressive disorder (MDD). However, few studies have reported the relationship between thyroid function, lipid metabolism and clinical profiles in female MDD patients. Thus, this study aimed to investigate the prevalence of psychotic depression (PD) and its risk factors in first-episode and drug naive (FEDN) depression among the female population in China.

**Methods:**

This was a cross-sectional study involving a representative probability sample of 1,130 FEDN female outpatients with MDD (aged 18 years or older) in China. We collected information relating to socio-demographic characteristics, clinical data and blood samples. The Hamilton Depression Rating Scale 17-item version (HAMD-17), Hamilton Anxiety Rating Scale 14-item version (HAMA-14), and Positive and Negative Syndrome Scale (PANSS) were used to evaluate depressive, anxiety, and psychotic symptoms.

**Results:**

The prevalence of psychotic symptoms in female MDD patients was 10.97%. The findings revealed significant differences between MDD female patients with psychotic symptoms and non-PD female patients in the following areas: higher HAMD scores, higher HAMA scores, more severe anxiety and an increased risk of suicide attempts. Further logistic regression analysis showed that psychotic symptoms were associated with higher thyroid-stimulating hormone (TSH) levels and an odds ratio of 1.168.

**Conclusions:**

Our findings supported the hypothesis that higher TSH levels were correlated with psychotic symptoms in female MDD patients. Therefore, serum TSH levels may be a potential biomarker of PD in female MDD patients. In addition, we found that PD was closely associated with suicide attempts and lipid levels, but did not reach statistical significance.

## Background

Major depressive disorder (MDD) is a common and disabling psychiatric disorder that severely diminishes quality of life and impairs social function, resulting in sleep disturbance, anxiety symptoms, anhedonia, weight loss, psychomotor retardation, and even disability [1, 2]. Globally, it accounts for 4.46% of disability-adjusted life-years (DALYs) and 12.1% of years lived with disability (YLD), remaining one of the leading causes of disability and disease burden for both men and women [3]. Furthermore, approximately 58% of MDD patients attempt suicide and the standard mortality ratio (SMR) of suicide has risen to 20.4%, which is the leading cause of mortality in MDD patients [4].

Psychotic depression (PD), a severe form of MDD, has been found 18.5% of MDD patients meet the criteria for psychotic features [5]. PD has been found to always occur alongside severe cognitive dysfunction and increased suicide attempts [6]. Concordantly, Dold M suggested that PD was associated with more severe depressive symptoms, a higher risk of suicide and increased treatment resistance in comparison to non-psychotic MDD patients [7]. Compared with non-PD patients, the mortality rate among PD patients is as high as 41%, which is significantly higher than the rate observed among non-PD patients, at 20%[8]. On the other hand, several experimental studies have found no association between suicidality and psychotic features in MDD patients [9]. Therefore, the results are inconsistent and the mechanisms remain unclear.

The relationship between sex and MDD has been heavily demonstrated over the past few years. Globally, in women, depression is the fourth main cause of disease burden whereas it ranks seventh among men [10]. Compared to men, a greater association has been found between females with MDD and lifelong suicidal ideation and suicide attempts [11]. Differences in genetics and biological factors involving hormones and neuroticism may explain the sex gap that is found in the case of depression [12]. In addition, female patients with PD are at a higher risk of fatigue, mood-inconsistent delusions, and anxiety disorders than male patients, according to previous research that concentrated on sex differences in MDD with psychotic symptoms [13]. Thus, there is a need to carry out a more comprehensive investigation of females with MDD and psychotic symptoms.

Recently, several studies have pointed to biomarkers in thyroid function and lipid metabolism in MDD patients with psychotic symptoms [14]. However, the findings of various studies are conflicting. For example, some earlier studies reported a possible interaction between the mechanisms of thyroid hormones and psychotic behaviors [15, 16]. Moreover, in some recent studies, a correlation has been found between lipid levels and psychotic depression [17]. However, other studies have failed to support these correlations [18]. Currently, accumulating evidence has found that abnormal levels of thyroid-stimulating hormone (TSH) are associated with a high risk of depressive symptoms in women [19]. Concordantly, this was found to be more common in dyslipidemia and in increased triglycerides (TG) levels among women with MDD who take antidepressant medication, which was not found in men [20]. In conclusion, it is necessary to explore the possible relationships between thyroid hormone and lipid levels in female MDD patients having psychotic features.

Moreover, various studies have suggested that different countries and cultures significantly affect the emergence of psychotic symptoms; for example, the burden rate of depression is 4.1% in developing countries and has risen to 8.9% in developed countries [10, 11, 21]. However, few studies on MDD have investigated the factors related to psychotic symptoms in women from among the Chinese population. Therefore, it is necessary to study the relationship between biochemical indicators and the clinical correlates of psychotic symptoms in Chinese women with MDD. Furthermore, our study focused on the Han population, and the large sample size included first-episode and drug naïve (FEDN) female MDD patients. This study aimed to explore the correlations between clinical profiles and related factors (e.g., suicide attempts, thyroid function, lipid levels and blood glucose) in FEDN female MDD patients in the Chinese population.

## Methods/participants

### Subjects

This cross-sectional study was conducted at the First Clinical Medical College of Shanxi Medical University in Shanxi Province, China. From 2015 to 2017, 1,130 female patients participated in the study and voluntarily signed an official consent form.

In this study, all patients satisfied the following inclusion criteria: (1) females aged 18 years or older of Han nationality. (2) first-episode patients without any previous history of medication or treatment. (3) satisfied the MDD criteria based on the Diagnostic and Statistical Manual of Mental Disorders, Fourth Edition (DSM-IV), and diagnosed by two clinical psychiatrists.

The exclusion criteria included: (1) history of other psychiatric diagnosis other than MDD (as determined by DSM-IV), such as bipolar disorder or alcohol or drug dependence. (2) diagnosed with other severe physical diseases. (3) pregnant or breastfeeding women. (4) refusal to provide written consent form to participate in this study. (5) any apparent thyroid disease in the past or current medical history.

### Socio-demographic characteristics and clinical measures

Socio-demographic data included age, education, marital status and body mass index (BMI).

Clinical measures included duration of untreated illness (DUI) and age of onset. To ensure the credibility of evaluations across the study, the Hamilton Depression Rating Scale 17-item version [22] (HAMD-17), Hamilton Anxiety Rating Scale 14-item version [23] (HAMA-14) and Positive and Negative Syndrome Scale (PANSS) [24] were used. The HAMD-17 was utilized to assess depressive symptoms. Depressed female patients with HAMD-17 scores higher than 24 were assigned to the severe depressive symptoms group. The HAMA-14 was used to measure anxiety symptoms. HAMA-14 scores higher than 28 were classified as severe anxiety symptoms. The PANSS was employed to evaluate psychotic symptoms. MDD patients were considered to have psychotic symptoms if they scored 15 or above on the positive symptom subscale. These scales are widely used in China and have a certain reliability. The inter-rater correlation coefficient of HAMD, HAMA, and PANSS scores exceed 0.8.

A suicide attempt was defined as a provable self-injurious behavior wherein the individual was determined to end their own life [25]. All participants were asked the same question: “Have you ever attempted suicide in your whole life?“ If they answered “yes”, they were considered to be suicide attempters. Then, interviews were conducted to obtain more information based on the following questions: " When, where, how, and how many times did you attempt suicide?“ to obtain information about the date, place, method, and frequency of suicide. If they could not remember the details and their answers were not clear, got more information from their family members, relatives, or friends.

### Collection and detection of blood samples and others

Serum samples were collected between 6 a.m. and 8 a.m. after overnight fasting and measured before 11 a.m. on the same day in the laboratory center of the hospital. The serum levels of thyroid hormone including TSH, anti-thyroglobulin antibodies (TGAb), antithyroid peroxidase autoantibody (TPOAb), free triiodothyronine (FT3) and free thyroxine (FT4) were measured by chemiluminescence immunoassay. The following fasting serum indexes were measured: fasting blood-glucose (FBG), total cholesterol (TC), triglyceride (TG), high-density lipoprotein cholesterol (HDL-C), and low-density lipoprotein cholesterol (LDL-C). Furthermore, in MDD patients, levels of systolic blood pressure (SBP) and diastolic blood pressure (DBP) were measured.

### Statistical analysis

In order to compare differences in the demographic, clinical and biochemical characteristics and parameters relating to the PD group, we performed a one-way analysis of variance (ANOVA) for continuous variables and a chi-square test for categorical variables. Furthermore, a binary logistic regression analysis was carried out to analyze the variables which were most associated with PD. Odds ratios (ORs) and 95% confidence intervals (95% CIs) were computed. In the PD group, we used two models (without TSH or without TC) to investigate the risk factors because of the strong correlation between TSH and TC value. Moreover, Bonferroni correction was applied to adjust for multiple testing. All tests were two-sided and a p-value lower than 0.05 was deemed statistically significant. Statistical analyses were carried out with SPSS version 24.0 (IBM, Chicago, IL, USA).

## Results

### Demographic, clinical characteristics and biochemical parameters of recruited subjects

A total of 1,130 female patients were enrolled in this study, of whom 124 (11.0%) complied with the standard for psychotic symptoms and 1006 (89.0%) did not. Among female MDD patients, a significant difference was observed between the PD group and non-PD group with respect to level of education. Furthermore, significantly higher total HAMD (33.72 ± 2.471 vs. 29.88 ± 2.712) and HAMA (26.33 ± 3.001 vs. 20.20 ± 2.945) (both p < 0.05) scores were observed in PD group. Subjects with accompanying psychotic symptoms had higher serum levels of TSH, TgAb, TPOAb, FBG, TC, TG and LDL-C and lower levels of HDL-C (all p < 0.05). Moreover, levels of systolic and diastolic blood pressure in the PD group were significantly higher than in the non-PD group (Table 1).

**Table 1:** Demographic, clinical characteristics and biochemical parameters between PD and non-PD female patients

Variable	MDD without PD (n = 1,006)		MDD with PD (n = 124)		*χ2/F*	*p*
Age, mean (SD)	35.55	12.270	37.60	13.714	7.84	0.114
DUI, mean (SD)	6.291	4.652	6.742	4.764	0.478	0.310
Age of onset, years	35.35	12.158	37.39	13.606	8.078	0.113
Education					9.061	0.028*
Junior high school, n (%)	243	24.2	45	36.3		
High school, n (%)	440	43.7	49	39.5		
University degree, n (%)	255	25.3	24	19.4		
Master’s degree, n (%)	68	6.8	6	4.8		
Marital status					0.008	0.928
Single	272	27	34	27.4		
Married	734	73	90	72.6		
HAMD, mean (SD)	29.88	2.712	33.72	2.471	2.519	<0.001***
HAMA, mean (SD)	20.20	2.945	26.33	3.001	0.005	<0.001***
Suicide attempt	171	17.0	63	50.8	76.847	<0.001***
Comorbid anxiety	54	5.4	90	72.6	448.501	<0.001***
TSH, uIU/mL	4.828	2.365	7.606	3.092	28.350	<0.001***
TGAb, IU/L	83.983	239.936	169.614	364.483	28.337	0.012*
TPOAb, IU/L	65.946	152.948	130.541	245.738	35.497	0.005**
FT3, pmol/L	4.878	0.730	4.888	0.654	3.065	0.888
FT4, pmol/L	16.718	3.085	16.479	2.944	0.150	0.414
FBG, mmol/L	5.376	0.625	5.695	0.756	7.843	<0.001***
TC, mmol/L	5.202	1.1049	5.772	1.177	1.180	<0.001***
TG, mmol/L	2.144	0.981	2.459	0.931	1.091	0.001**
HDL-C, mmol/L	1.229	0.284	1.124	0.261	0.653	<0.001***
LDL-C, mmol/L	2.966	0.866	3.230	0.834	1.018	0.001**
BMI, kg/m2	24.313	1.856	24.615	1.911	0.172	0.089
Systolic BP, mmHg	119.17	10.885	125.43	11.821	1.481	<0.001***
Diastolic BP, mmHg	75.61	6.486	78.94	7.378	3.352	<0.001***

**NOTE**: MDD: major depressive disorder; DUI: duration of untreated illness; HAMD: Hamilton depression rating scale; HAMA: Hamilton anxiety rating scale; TSH: thyroid-stimulating hormone; TGAb: anti-thyroglobulin antibodies; TPOAb: antithyroid peroxidase autoantibody; FT3: free triiodothyronine; FT4: free thyroxine; FBG: fasting blood-glucose; TC: total cholesterol; TG: triglyceride; HDL-C: high-density lipoprotein cholesterol; LDL-C: low-density lipoprotein cholesterol; BMI: body mass index

*p < 0.05; **p < 0.01; ***p < 0.001

### The risk factors for suicide attempts and PD in female MDD patients

The correlation analysis revealed a significant association between TSH serum level and TC serum level (r = 0.56, p < 0.001). Therefore, two binary logistic regression models (without TSH or without TC) were used to analyze relational risk factors. Risk factors related to PD are shown in Table 2. In Model 1, female MDD patients with psychotic symptoms showed significant differences with respect to the following: HAMD scores (B = 0.257, OR = 1.293, 95% CI = 1.136 − 1.472), HAMA scores (B = 0.357, OR = 1.429, 95% CI = 1.208 − 1.689), comorbid anxiety (B = 1.216, OR = 3.357, 95% CI = 1.384 − 8.230) and TSH levels (B = 0.156, OR = 1.168, 95% CI = 1.014 − 1.346). On the other hand, in Model 2, significant differences in the PD group were only observed with respect to HAMD scores (B = 0.277, OR = 1.319, 95% CI = 1.156 − 1.505), HAMA scores (B = 0.373, OR = 1.452, 95% CI = 1.231 − 1.712) and comorbid anxiety (B = 1.213, OR = 3.364, 95% CI = 1.389 − 8.143), but with no difference in TC levels.

**Table 2:** Model 1: Risk factors (without TC) associated with psychotic symptoms of adjusted odds ratio in female MDD patients. Model 2: Risk factors (without TSH) associated with psychotic symptoms of adjusted odds ratio in female MDD patients

	Model 1					Model 2					
	B	Wald statistic	p	OR	95% CI	B	Wald statistic	P	OR	95%CI	
Education	-0.298	3.242	0.072	0.743	0.537 − 1.027	-0.278	2.848	0.091	0.757	0.548–1.046	
HAMD	0.257	15.107	<0.001***	1.293	1.136 − 1.472	0.277	16.899	<0.001***	1.319	1.156–1.505	
HAMA	0.357	17.400	<0.001***	1.429	1.208 − 1.689	0.373	19.625	<0.001***	1.452	1.231–1.712	
Suicide attempt	-0.406	1.644	0.200	0.667	0.359 − 1.239	-0.276	0.775	0.379	0.759	0.411–1.402	
Comorbid anxiety	1.216	7.152	0.007**	3.357	1.384 − 8.230	1.213	7.230	0.007**	3.364	1.389–8.143	
TgAb	0.000	0.011	0.917	1.000	0.999 − 1.001	0.000	0.088	0.766	1.000	0.999–1.001	
TPOAb	0.001	0.934	0.334	1.001	0.999 − 1.002	0.001	1.253	0.263	1.001	0.999–1.002	
FBG	-0.011	0.002	0.960	0.989	0.645 − 1.517	0.215	1.084	0.298	1.240	0.827–1.858	
TG	0.149	1.097	0.295	1.160	0.879 − 1.532	0.234	2.587	0.108	1.263	0.950–1.679	
HDL-C	0.041	0.007	0.935	1.042	0.392 − 2.771	-0.351	0.482	0.488	0.704	0.261–1.899	
LDL-C	-0.253	2.305	0.129	0.776	0.560 − 1.076	-0.072	0.144	0.705	0.931	0.642–1.349	
Systolic BP	-0.016	0.670	0.413	0.984	0.948 − 1.022	0.000	0.000	0.983	1.000	0.964–1.036	
Diastolic BP	0.009	0.108	0.742	1.009	0.955 − 1.067	0.009	0.100	0.752	1.009	0.955–1.066	
TSH	0.156	4.657	0.031*	1.168	1.014 − 1.346						
TC						-0.237	1.894	0.169	0.789	0.563–1.106	

*p < 0.05; **p < 0.01; ***p < 0.001

## Discussion

This study found that female MDD patients with PD had lower education levels and higher HAMD and HAMA scores compared to female MDD patients without PD. This study also found that comorbid anxiety and suicide attempts were associated with psychotic symptoms in female MDD patients. Also, our study identified some associations between thyroid function and psychotic symptoms in Chinese female outpatients with MDD.

This study revealed that the prevalence of PD among FEDN female patients was 11.0%. Meanwhile, the prevalence rate of psychotic features in MDD was 10.92% in European countries [7]. In Gaudiano BA’s study, about 13.2% of MDD patients had psychotic features [26], which was similar to our findings. However, some studies produced completely different results, and found that the prevalence of PD was 35.0% among MDD inpatients at a Denmark psychiatric hospital [27]. In a Thai study, approximately 35.3% of outpatients with MDD had one or more psychiatric comorbidity [28]. The large discrepancy among the studies may be attributed to following: First, the data from the surveys showed dramatic differences with respect to hospitalized patients and outpatients. The sample in the European study, Gaudiano BA’s study and our study included outpatients, whereas the Danish data were derived from inpatients. Second, different countries and regions may produce different results because of the influence of race, religion, social characteristics, education level, variations in diagnostic protocol and differences in mental health services [3].

From the HAMD and HAMA psychotic symptom subscale scores, significant differences were observed between female patients with PD and those without psychotic symptoms. Therefore, we speculated that higher HAMD and HAMA subscales scores accompany more serious levels of anxiety and fear [29]. Interestingly, our study also showed that comorbid anxiety had a significant impact on the presence of psychotic symptoms in female MDD patients, with ORs of 3.357 (in Model 1) and 3.364 (in Model 2), which was consistent with a recent study that revealed higher anxiety scores in MDD patients with PD [30]. Several studies pointed out that anxiety and depression increase the responses to social stress, further stimulating the hypothalamic-pituitary-adrenal (HPA) axis [31], leading to a higher level of corticotropin-releasing hormone (CRH) and a rise glucocorticoid concentrations. In this way, the higher glucocorticoid level leads to higher levels of stress and tension, which is associated with higher HAMD and HAMA scores and the presence of psychotic features.

Another finding of our study was that PD was significantly associated with suicide attempts in female MDD patients, and the PD group had a higher risk of suicide behaviors. Most previous studies were consistent with this result. For example, one study reported that the presence of psychotic symptoms can exacerbate the level of depression and further increase the risk of lifetime suicide attempts in adolescent MDD patients [32]. We hypothesized that patients who exhibit more psychotic symptoms also tend to experience increased anxiety and depressive symptoms [33], which may be a possible risk factor for suicide attempts. However, some studies had conflicting views. A previous study showed that MDD patients with additional psychiatric disorders did not show an increased risk of suicide [34]. Another recent research failed to find a significant association between psychotic symptoms and suicide attempts in patients with MDD [35]. A number of possible reasons may account for these discrepancies: First, different age groups may produce different results due to physiological and psychosocial changes. Second, different ethnic populations may influence the results due to different difference social and cultural characteristics. Third, study samples may comprise either inpatients or outpatients, which may produce conflicting results. In addition, our study found that PD may increase the risk of suicide attempts; however, it did not reach statistical significance (p = 0.2 in Model 1 and p = 0.379 in Model 2).

The association between abnormal thyroid hormones (mainly TSH) and female MDD patients with psychotic symptoms was also a significant finding in our study. A retrospective study of TSH measurements in subjects with non-thyroidal disease found that females had higher TSH and TPOAb values than males in all age groups. Moreover, TSH levels increased with age [36, 37]. Meanwhile, in some earlier research studies, serum TSH levels in the upper 25th percentile of the normal reference range were closely associated with more severe depression symptoms, a higher risk of suicide attempts and stronger treatments for major depression [38]. Thus, we were able to hypothesize that the high level of TSH in women is associated with anxiety [39] and positive symptoms of depression, and is further correlated with psychotic symptoms. Interestingly, in our study, the PD group had higher TPOAb levels compared to the non-PD group, which is similar to the findings of Carta MG; there was also a correlation between higher TPOAb values and both mood and anxiety disorder [40]. However, in further logistic analysis, serum TPOAb, FT4 and FT3 levels showed no significant differences between PD and non-PD groups. In conclusion, it is necessary to explore the relationships and possible mechanisms between thyroid hormones and psychotic symptoms.

Our study found that lipid levels were significantly higher in female patients with psychotic symptoms. From Ledochowski’s data, we can see that higher cholesterol concentrations were correlated with an increased susceptibility for depression [41]. Thus, we speculate that patients with depressive mood disorders may have a tendency to increase their carbohydrate intake, which contributes to higher cholesterol levels [42]. In this way, there were some uncertainties about whether the higher TC levels were the result of or the reason for the patients’ depression. Moreover, our finding that higher TC levels were associated with psychotic symptoms in female MDD patients was consistent with previous research results. A study in Taiwan showed that, compared with healthy subjects, patients with severe psychiatric symptoms always occurred alongside higher serum cholesterol levels [43]. However, some studies held distinct views about this; lower levels of TC were associated with severity of MDD and psychotic symptoms [44, 45]. The inconsistency of these results may be due to the following reasons: First, these studies included subjects of various ages, but the prevalence of lipid and glucose abnormalities was higher in those aged older than 40 years [46]. Second, some studies investigated bipolar depression or used a small sample size, which may have contributed to some inaccuracy. Furthermore, a study reported that female MDD patients had a higher risk of lipid metabolism disorder [46], and Meng’s study demonstrated that women were prone to hypertriglyceridemia if their TSH levels were above 4.0 mIU/mL [47], which can increase the risk of psychotic symptoms. In addition, there were several possible mechanisms linking abnormal lipid levels to female MDD patients with PD, namely, the HPA axis [45], inflammation [47] and the serotonin (5HT) system [48], although research studies in these areas were inconsistent and the mechanisms were unclear.

Several limitations need to be noted in our current study: First, this was a cross-sectional study without long-term follow-up data. Therefore, further study is needed to elaborate upon the relationships between comorbid anxiety, suicide attempts, thyroid dysfunction and lipid levels in female MDD patients with psychotic symptoms. Second, age and sex had a significant effect on hormone levels and metabolism disorders, but our study included women with a large age range (18 ~ 60 years) and excluded pregnant or lactating women. A retrospective and cross-sectional study pointed out that women over 40 years of age had a particularly high incidence of lipid and glucose abnormalities [46]. Thus, our study’s inclusion and exclusion criteria might have caused bias with respect to hormone levels and metabolism disorders. Third, the subjects in this study were outpatients, and it was preferable to include inpatients and community patients in order to produce more accurate data. Fourth, the variables related to suicide were not comparatively detailed, such as history and severity of suicide. Fifth, it would be more appropriate to use a specific scale to evaluate psychotic depression; for example, the Psychotic Depression Assessment Scale (PDAS) could be used instead of the PANSS, so as to obtain more reliable results on the severity of psychotic symptoms. Sixth, there are challenges in diagnosing first-episode patients and patients with bipolar disorder, and we cannot be certain that all samples of female MDD patients will not undergo a diagnostic conversion to bipolar depression. Therefore, in the future, we should follow all patients over time to minimize any bias. Finally, all 1130 patients in this study were from a particular region of China. In the future, we will conduct further multi-center studies to explore and validate our findings more precisely.

In conclusion, our study found a significant relationship between anxiety and psychotic symptoms in female MDD patients. More importantly, higher levels of TSH could reflect thyroid dysfunction and be correlated with psychotic symptoms. Thus, serum TSH levels may be a possible biomarker of PD in female MDD patients, although the specific mechanism has yet to be explored. Furthermore, we found that PD was closely associated with suicide attempts and lipid levels, but it did not reach statistical significance. In future clinical management, it would be helpful to instruct clinicians to reduce the prevalence of psychotic symptoms in female patients with MDD by decreasing anxiety and monitoring abnormal serum thyroid levels.

## Data Availability

The data are available from the corresponding author on reasonable request.

## List of abbreviations

MDD: Major depressive disorder
PD: Psychotic depression
FEDN: First-episode and drug naïve
HAMD-17: Hamilton Depression Rating Scale 17-item version
HAMA-14: Hamilton Anxiety Rating Scale 14-item version
PANSS: Positive and Negative Syndrome Scale
DALYs: Disability-adjusted life-years
YLD: Years lived with disability
SMR: Standard mortality ratio
DSM-IV: Diagnostic and Statistical Manual of Mental Disorders, Fourth Edition
BMI: Body mass index
TSH: Thyroid-stimulating hormone
TGAb: Anti-thyroglobulin antibodies
TPOAb: Antithyroid peroxidase autoantibody
FT3: Free triiodothyronine
FT4: Free thyroxine
FBG: Fasting blood-glucose
TG: Triglycerides
TC: Total cholesterol
HDL-C: High-density lipoprotein cholesterol
LDL-C: Low-density lipoprotein cholesterol
SBP: Systolic blood pressure
DBP: Diastolic blood pressure
